# Protective efficacy of combined trivalent inactivated ISA 71 oil adjuvant vaccine against avian influenza virus subtypes (H9N2 and H5N1) and Newcastle disease virus

**DOI:** 10.14202/vetworld.2017.1212-1220

**Published:** 2017-10-11

**Authors:** Zeinab Mohamed Ali, Mervat Abd El Monaem Hassan, Hussein Ali Hussein, Basem Mohamed Ahmed, Ahmed Abd El-Ghany El Sanousi

**Affiliations:** 1 Department of Poultry Vaccines, Production Unit Veterinary Serum and Vaccine Research Institute, Abbasia 11759, Egypt; 2 Department of Virology, Faculty of Veterinary Medicine, Cairo University, Giza 12211, Egypt

**Keywords:** avian influenza, immunization, Newcastle disease virus, shedding, trivalent vaccine

## Abstract

**Aim:**

The objective of the present study was to prepare a trivalent inactivated vaccine of Newcastle disease virus (NDV), H5N1, and H9N2 viruses.

**Materials and Methods:**

Three monovalent and a trivalent vaccines were prepared by emulsifying inactivated NDV (LaSota strain), reassortant H5N1, and H9N2 viruses with Montanide ISA 71 oil adjuvant. Parameters used for evaluation of the efficacy of the prepared vaccines in specific pathogen-free chickens were cellular immunity assays (blastogenesis, interferon gamma, interleukin 1 [IL1], and IL6), humoral immunity by hemagglutination inhibition, protection percentage, and shedding.

**Results:**

A single immunization with trivalent vaccine-enhanced cell-mediated immunity as well as humoral immune response with 90% protection against challenges with highly pathogenic avian influenza (HPAI) H5N1 and low pathogenic (LP) avian influenza H9N2 viruses with 100% protection after challenge with NDV.

**Conclusion:**

Development and evaluation of the trivalent vaccine in the study reported the success in preparation of a potent and efficacious trivalent vaccine which is a promising approach for controlling HPAI H5N1, LP H9N2, and ND viral infections.

## Introduction

Avian influenza virus (AIV) belongs to the Orthomyxovirus family. AIV infections can cause various disease symptoms in chickens, ranging from asymptomatic infection to respiratory disease, accompanied with reduced egg production and/or severe systemic diseases with near 100% mortality rates. The severity of the disease in poultry is determined by genetic features where the infection is classified as either low pathogenic (LP) avian influenza (LPAI) or highly pathogenic avian influenza (HPAI) [1]. Evidence suggests that virus elimination in poultry is improbable in a few countries where the virus remains endemic. In these endemic countries such as Bangladesh, China, Egypt, India, Indonesia, and Vietnam, all accessible tools for prevention and control of the disease should be considered, including vaccination using suitable and quality biologicals [2]. Production of a safe and high-yield H5N1 vaccine strain is challenging for vaccine manufacturers; therefore, the reverse genetics system, which uses a high-growth backbone virus, offers a key for the generation of a high-yield, avirulent influenza vaccine strains for vaccination of poultry species [3,4]. It has been reported that as the quantity of AIV antigen in the vaccines increases, all parameters of protection improve, which is strain dependent [5].

AIV subtype H9N2 is categorized as LPAI virus (LPAIV), but it can cause serious economic losses in poultry industry including reduced egg production and decreased growth rate. Moreover, it can occasionally cross the species barrier and cause human infections, which has raised public health concerns. In February 2015, the first human case of H9N2 subtype virus infection in Egypt was reported [6]. This event compelled national and international authorities to examine the reasons behind the increase in human infections and implement control measures [6]. Coinfection of H9N2 with H5N1 was also reported in many cases in poultry in Egypt [7-10]. Although predictable, reassortment between H5N1 and H9N2 has not been yet reported [11]. H9N2 vaccination has been used to face the field outbreaks [12]. A bivalent mucosal inactivated H9N2 and Newcastle disease virus (NDV) have already been established in the country [13].

ND caused by *Avian avulavirus* 1 (avian paramyxovirus serotype-1) is considered as one of the most overwhelming poultry infections, owing to its worldwide distribution and economic implications. NDVs have been categorized into lentogenic, mesogenic, and velogenic strains according to disease severity in chickens [14]. Live vaccines based on the lentogenic LaSota or other lentogenic strains are routinely applied to chicken and have been proved to induce high levels of immunogenicity and protective efficacy against lethal velogenic strains [15,16]. Practically, using these, virus vaccines separately stressful for both the worker and the bird. Handling of laying birds usually results in decreased production, and sometimes, severe egg peritonitis may occur also, and the labor expense can be partly offset by the use of polyvalent vaccines. Inactivated oil-emulsion vaccines are not as badly affected by maternal immunity as live vaccines and can be used in day-old chicks. In this study, we developed a trivalent vaccine containing the inactivated NDV LaSota strain antigen, reassortant H5N1, and LPAI H9N2 virus antigens for vaccination in poultry. We also evaluated its immunogenicity and protective efficacy against lethal HPAI H5N1, virulent NDV virus infection, and LPAI H9N2 infection.

## Materials and Methods

### Ethical approval

Animal experiments were conducted in accordance with the guidelines laid down by the International Animal Ethics Committee and in accordance with the local laws and regulations.

### Vaccine preparation

#### Viruses’ propagation and titration

Vaccine strains and seed viruses were propagated in specific pathogen-free (SPF) embryonated chicken egg (ECE) [17,18] for H9N2, reassortant H5N1 viruses [18], and for Lasota NDV [19]. The obtained harvest from each virus was titrated in SPF ECEs and calculated according to a method of Read and Muench [17].

#### Avian influenza (AI) H9N2 master seed virus

The LP (A/chicken/Egypt/114922v/2011 [H9N2]), with accession Number (JQ419502), virus was provided by the National Laboratory for Quality Control on Poultry Production, Animal Health Research Institute, Dokki, Egypt. The virus was used for preparation of the vaccine seed virus. The original titer of the virus was 10^9.5^ egg infective dose (EID) 50/ml with hemagglutination (HA) activity of 10 Log2.

#### AI H5N1 master seed virus

Two reassortant AIVs (A/Chicken/Egypt/Q1995D/2010 [H5N1]) with a titer 10^10^ EID 50/ml and 10 Log2 HA activity and A/Duck/Egypt/M2583D/2010 (H5N1) of a titer 10^11^ EID 50/ml and 11 Log2 HA activity were used. These viruses were generated in the National Research Center, Giza, Egypt, and provided to the Veterinary Serum and Vaccine Research Institute, Newcastle Disease Unit, Abbasia, Cairo, Egypt.

#### Newcastle disease master seed virus

Lasota strain of NDV (lentogenic) was supplied by the Central Veterinary laboratories, New Haw, Weighbridge, Surry, UK. The virus was propagated in SPF chicken eggs. The allanto-amniotic fluids were harvested, dispensed in vials, lyophilized, and stored at −70°C. The original titer of the virus was 10^10.5^ EID 50/ml with HA activity 10 Log2.

#### SPF ECEs

Eggs were obtained from Nile SPF Farm, Kom Oshiem, Fayom, Egypt, and used for virus propagation, virus titration, and assurance of complete inactivation.

#### Inactivation of viruses

Inactivation of AI subtypes H9N2 and reassortant H5N1 and NDV viruses was carried out using formalin in a final concentration of 0.1% of the total volume. The fluid was blended using magnetic stirrer for about 20 h at 25°C. Sodium bisulfite was added as a final concentration of 2% to stop the action of formalin [20]. Samples from each inactivated virus were tested for complete inactivation in 10-day-old SPF ECE for two successive blind passages before it was considered free from residual live virus.

#### Antigen emulsification

Four vaccines were prepared (monovalent inactivated H9N2, H5N1, and NDV and a combined trivalent vaccine containing H9N2, H5N1, and NDV) as oil adjuvant vaccines using Montanide™ ISA 71 VG adjuvant (SEPPIC France) as per the manufacturer’s instructions.

### Vaccine evaluation

#### Safety test

An experimental batch of the prepared vaccine was tested for its safety by inoculating double dose subcutaneously in 10 3-week-old birds, and these are observed for 2 weeks for the presence of clinical signs of disease or local lesions [18].

#### Sterility test

An experimental batch of the prepared vaccine candidate was tested for sterility and freedom from any fungal or bacterial contaminants by culturing on specific media [18].

### Potency of prepared vaccines

A total of 250 1-day-old SPF chicks were purchased from Kom Oshiem SPF Farm, Fayoum, Egypt. The chicks were divided into five groups: Group 1 injected with monovalent H9N2, Group 2 for monovalent H5N1, Group 3 for monovalent NDV vaccine, Group 4 for trivalent vaccine (all chickens injected with 0.5 ml I/M of previously prepared vaccines), and Group 5 kept as non-vaccinated control group. Chickens housed in isolation facilities till they became 21 days of age with free access to water and feed.

#### Evaluation of cellular immune response

Heparinized blood samples were collected from the five groups at 3^rd^, 5^th^, 7^th^, 15^th^, and 21^st^ days postvaccination for lymphocyte proliferation assay and at 5^th^, 10^th^, 15^th^, and 21^st^ postvaccination for identification of interleukin 1 (IL1), IL6, and interferon gamma (IFN γ) genes by real-time polymerase chain reaction (PCR).

#### Evaluation of humoral immune response using HA inhibition (HI)

It was carried out using 4 HAU of homologous antigen (H9N2 AIV, H5N1, and NDV Lasota strain), to estimate antibody titers in sera of vaccinated and unvaccinated chickens [21].

#### Evaluation of vaccine protection and viral shedding

Challenge with viscerotropic velogenic NDV (VVNDV)

Ten birds were chosen randomly from trivalent vaccine-vaccinated group and control unvaccinated group were subjected to challenge test against NDV using the VVND [22], each bird received a dose of 0.5 ml I/M from the virulent VVNDV strain (10^6^ EID 50/ml) and observed for 15 days after challenge. Birds which died within this period were collected for a detailed P.M. examination for any characteristic lesions.

Protection % against VVNDV=Number of survivals/total number of challenged birds×100

Challenge with H9N2 LPAIV

Twenty chicks from the vaccinated and non-vaccinated groups were challenged with the LPAI A/chicken/Egypt/114922v/2011 (H9N2) at 30-day postvaccination. The birds were inoculated through the intranasal route (100 µl/chick) of allantoic fluid containing 10^6^ EID 50 of the virus. Tracheal swabs were collected at 3,5 and 7 days post challenge (DPC) to determine the virus shedding.

Challenge with HPAI H5N1

SPF chicken groups were vaccinated at 4 weeks of age. At 28-day postvaccination, all birds were challenged intranasally by local Egyptian HPAI H5N1 isolates (A\chicken\Egypt\VSVRI\2009). The challenge virus dose was 0.1 ml containing 5.5×10^5^ EID 50. Another group of chicks were kept as control unvaccinated and challenged with the same dose of the challenge virus. Birds were observed daily for 15 DPC. Three DPC, the morbidity and mortality rates were recorded for each group till the end of the observation period to measure the protection %. Tracheal swabs were collected at 3,5 and 7 days DPC to determine the virus shedding.

### Statistical analysis

Using computer software SPSS version 22.0 [23], simple one-way ANOVA was used to study lymphocyte blastogenesis assay and HI test, and Duncan’s multiple range tests were used to differentiate between significant mean [24]. The recorded data of cytokines (IL1, IL6, and IFN γ) were analyzed using two-sided Fisher’s exact test, and p<0.05 was considered as statistically significant.

## Results

### Sterility and safety of the prepared vaccines

All the four vaccine candidates were found to be sterile and safe in vaccinated birds, where they induced neither any bacterial or fungal growth nor any abnormal clinical signs.

### Lymphocyte blastogenesis

**Figure-1:**
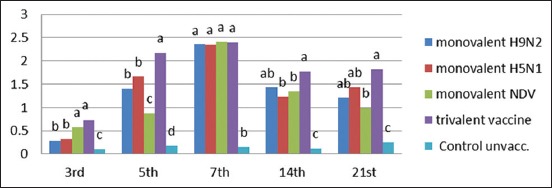
Lymphocyte blastogenesis assay using 2,3-bis(2-methoxy-4-nitro-5-sulfophenyl)-2H-tetrazolium-5-carboxanilide reagent expressed by DOD means with different alphabetical (a, b, c, and d) within the column are significantly different at p<0.05 using Duncan’s multiple range test.

There was a significant increase in lymphocyte proliferation at the 3^rd^ day postvaccination in all vaccinated groups compared to the control unvaccinated group with a significant difference between monovalent NDV, trivalent vaccine, and monovalent H9N2 and H5N1 groups (Figure-1). The lymphocyte proliferation reaches to maximum at the 7^th^ day postvaccination with no significant difference between all vaccinated groups at this age. However, at 21-day postvaccination, there was a significant increase in the lymphocyte proliferation in the trivalent vaccine compared with other monovalent groups.

### IL1 response of vaccinated chickens

**Figure-2:**
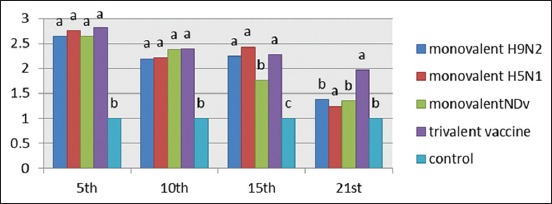
Interleukin-1 response after vaccination with 4 prepared vaccines at interval days postvaccination. The recorded data were analyzed using two-sided Fisher’s exact test, different alphabetical (a, b, c, and d) within the columns are significantly different at p<0.05 using Duncan’s multiple range test.

IL1 response of vaccinated groups of chicks at interval days as measured by quantitative reverse transcription (qRT)-PCR assay showed waves of increasing and gradual decreasing values differ from group to group as shown in Figure-2. There was no significant difference between the vaccinated groups at the 5^th^ and 10^th^ days postvaccination. At the 15^th^ day postvaccination, there was a significant difference in IL1 in group that received monovalent NDV vaccine compared to other groups. Meanwhile, at the 21^st^ day postvaccination, there was a significant increase in IL1 in a group of chicks vaccinated with trivalent vaccine.

### IL6 response of vaccinated chickens

Results of IL6 response in vaccinated chickens at interval days postvaccination showed no significant difference between the vaccinated groups at the 5^th^ and 10^th^ day postvaccination, while there was a significant difference between groups compared with internal control. At the 21^st^ day postvaccination, there was no significant increase in the vaccinated groups compared to internal control.

### IFN γ response of vaccinated chickens

Measuring the IFN γ response of vaccinated chicken groups by qRT-PCR assay showed increase in values at the 5^th^ and 10^th^ days postvaccination with no significant difference between vaccinated groups, while at the 15^th^ day postvaccination, there was a significant increase in IFN γ in group which received the trivalent vaccine. By 21^st^ day postvaccination, IFN γ began to decline with no significant difference between all groups and the internal control.

### Evaluation of humoral immune response

#### Monitoring of AI subtype H9N2 humoral immune response by HI test

**Figure-3:**
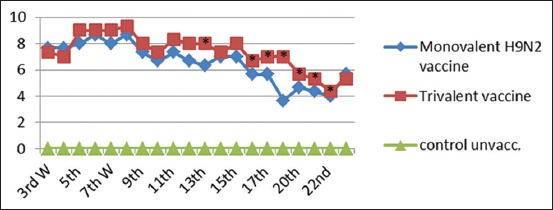
Mean hemagglutination inhibition antibody titers to H9N2 in vaccinated chickens with inactivated monovalent (H9N2) vaccine and trivalent (H5N1+H9N2+Newcastle disease virus). *Significant difference at p<0.05 using Duncan’s multiple range test.

It was noticed that chicks vaccinated with inactivated AI (H9N2) vaccine and the trivalent AI (H9N2-H5N1)+ND vaccine showed increased mean log2 HI antibody titer (7.67 Log2 and 7.33 Log2) from the 3^rd^ week postvaccination (WPV), respectively. The highest HI antibody titer (8.67 Log2 and 9.33 Log2) reached the 8^th^ WPV, and then, declined gradually to reach the lowest HI antibody titer (3.67 Log2 and 4.33 Log2) at the 24^th^ WPV for H9N2 monovalent vaccine and trivalent vaccine, respectively (Figure-3).

#### Monitoring of AI subtype H5N1 humoral immune response by HI test

It was noticed that chicks no significant difference in groups vaccinated with inactivated monovalent AI (H5N1) and trivalent vaccines with increased mean log2 HI antibody titers (7 Log2 and 7.67 Log2) from the 3^rd^ WPV, and then, reached the highest HI antibody titer (9 Log2) at the 8^th^ WPV, respectively. While at the 9^th^, 12^th^, 14^th^, 15^th^, 16^th^, 18^th^, and 22^nd^ WPV, there was a significant difference between the two vaccinated groups.

#### Monitoring of NDV humoral immune response by HI test

It was noticed that chicks vaccinated with inactivated Lasota NDV vaccine and trivalent AI (H9N2-H5N1)+ND vaccine showed increased mean log2 HI antibody titer (6.33 Log2 and 8.33 Log2) from the 3^rd^ WPV with significant difference between two groups at 3^rd^, 7^th^, 8^th^, 10^th^, 12^th^, 13^th^, 17^th^, 18^th^, and 22^nd^ WPV. The mean Log2 HI antibody titer reaches highest at the 8^th^ WPV (9 and 10.67 Log2) for monovalent NDV and trivalent vaccines, respectively, and then began to decline gradually (4 and 4.33 Log2 for monovalent NDV and trivalent vaccine, respectively) with no significant difference.

### Efficacy of the prepared vaccines (challenge and shedding)

#### Protective effectiveness of AI Type A H5N1 inactivated vaccine and combined vaccine against HPAI H5N1

All vaccinated chickens did not show any H5N1 symptoms post challenge while unvaccinated group showed typical HPAI H5N1 clinical and postmortem signs. The protection percent was 90% in both monovalent and trivalent vaccines (Table-1). Shedding test was carried out at 3, 5, and 7 DPC from oropharyngeal swabs revealed that both monovalent H5N1 and trivalent vaccines could reduce shedding of the H5N1 virus. Only one bird shed the virus 3 and 5 DPC. The shedding levels were 2 and 1 logs of EID 50/ml, respectively, for monovalent H5N1 with no viral shedding at 7 DPC. On group vaccinated with trivalent vaccine, only one bird shed H5N1 at 3 and 5 DPC. The shedding level was 1.5 and 1 logs of EID 50/ml, respectively, with no shedding virus at 7 DPC.

**Table-1:** Protective effectiveness of AI Type A H5N1 monovalent vaccine and trivalent vaccines against HPAI H5N1.

Chicken groups	Mean antibody titter in HI test (Log2)		Virus isolation from oropharyngeal (mean EID 50/ml)			Dead birds/total birds	Protection %
						–	
	1 WPC	2 WPC	3^rd^ DPC	5^th^ DPC	7^th^ DPC		
Monovalent H5N1	7	8	(1/10) 2	(1/10)=1.0	0/10	1/10	90
Trivalent vaccine	7.5	8	(1/10) 1.5	(1/10)=1.0	0/10	1/10	90
Control non vaccinated	0	0	0	N/A	N/A	10/10	0

WPC=Week post challenge, DPC=Days post challenge, N/A=Not applicable, EID=Egg infective dose, HI=Hemagglutination inhibition, AI=Avian influenza

The mean antibody titers at 1 and 2 weeks post challenge (WPC) were 7 and 8 Log2 in monovalent H5N1 vaccine and 7.5 and 8 Log2 in the trivalent vaccine groups, respectively.

#### Protective effectiveness of single H9N2 vaccine and combined vaccine against H9N2

The challenge test was carried out against LPAI H9N2 for both monovalent H9N2 and trivalent vaccines. Shedding test was carried out at 3, 5, and 7 DPC from oropharyngeal swab revealed that both monovalent H9N2 vaccine and combined trivalent vaccine were able to reduce the shedding of H9N2 virus that only one bird shed the virus 3 DPC, the shedding level was 1 EID 50/ml for monovalent H9N2 vaccine while there is no viral shedding at 5 and 7 DPC. In trivalent vaccine group, there was no viral shedding through the entire testing period. The mean HI antibody titer was 7.5 and 8 Log2 after the 1 and 2 WPC, respectively, in monovalent H5N1 vaccinated group, while in combined vaccine, the mean HI Ab titer was 7.7 and 9 Log2 at 1^st^ and 2^nd^ WPC, respectively. Compared to the control group, there was a viral shedding, and among the entire testing period, it was recorded as 5.5, 6, and 5 EID 50/ml at 3, 5, and 7 DPC, respectively, while there was no HI titer in the control group.

#### Protective effectiveness of single NDV vaccine and trivalent vaccine against challenge with virulent NDV

Challenge test was carried out against VVNDV for monovalent NDV and trivalent vaccines. All vaccinated chickens did not show any symptoms post challenge. Control non-vaccinated chickens showed typical clinical and postmortem signs of VVNDV infection. All non-vaccinated birds died after 5 DPC. This test reflects the protection percent induced by the prepared vaccine candidates as it was 100% in both monovalent NDV and trivalent vaccine. A rapid increase in the HI titer against NDV after the challenge was observed. The mean titer was 8 and 9.5 Log2 after 1^st^ and 2^nd^ WPC in monovalent vaccine while in trivalent vaccine the titers were 8.5 and 10.5 Log2 HI, respectively.

## Discussion

Polyvalent vaccine strategies increase reactivity for many pathogens including, but not limited to, influenza [25,26] although polyvalent vaccine formulations clearly expand the breadth of a single vaccine formulation, the reactivity is still limited to the individual components. The goal of polyvalency is to increase the breadth of vaccine coverage by combining diverse components into a single vaccination.

In this study, the obtained results revealed that all the prepared forms of ND and AI subtypes H5N1 and H9N2 vaccine candidates either monovalent or polyvalent were free from foreign contaminants and safe for vaccinating chickens which showed no detectable signs of illness as the recommendation of OIE [18]. The role for cell-mediated immunity in protection against AI virus is limited. T cells are the most important cells that mediate the cellular immune response, and the T cell subpopulations with diverse functions have been identified in chickens [27]. In this study, the cellular immune response in vaccinated and control groups was estimated using the lymphocyte proliferation test as well as cytokines (IL1, IL6, and IFN γ).

Analysis of the results of lymphocyte blastogenesis test Figure-1 revealed that all the vaccinated groups demonstrate cellular immune response with a significant increase (p<0.05) compared with the control unvaccinated groups. Similar observation was previously reported by El-Bagoury *et al*. [28] where chicken vaccinated with inactivated NDV ISA 71 vaccine induced higher cellular immune response as estimated by lymphocyte proliferation test.

**Figure-4:**
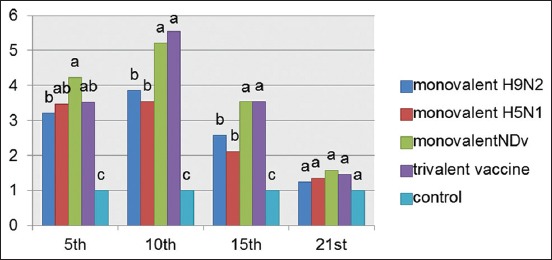
Interleukin-6 response in chicken vaccinated with four types of vaccines at interval days postvaccination. The recorded data were analyzed using two-sided Fisher exact test, different alphabetical (a, b, c, and d) within the columns are significantly, different at p<0.05 using Duncan’s multiple range test.

**Figure-5:**
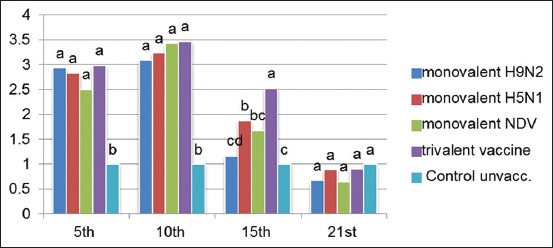
Interferon gamma response in vaccinated chickens at interval days postvaccination. The recorded data were analyzed using two-sided Fisher’s exact test, different alphabetical (a, b, c, and d) within the columns are significantly different at p<0.05 using Duncan’s multiple range test.

Using qPCR to characterize the expression of IL1B, IFN γ, and IL6 genes to provide insights into the role of innate immune response in protection against NDV and AIV subtypes H5N1 and H9N2 infection, results of cytokines (IL1b, IL6, and IFN γ) genes expression showed the presence of upregulation of the three genes with marked increase in IL6 gene expression in all types of prepared vaccines compared to the internal control of chickens 5-day postvaccination (Figures-2,4,5).

IL1 response of vaccinated groups of chicks at specific intervals showed waves of increasing and gradually decreasing values differing from group to group (Figure-2) where the highest value at the 5^th^ day postvaccination and then decreased gradually. On the 21^st^ day postvaccination, Group 4 which received combined trivalent vaccine showed superior value. IL1 production would be expected in many avian infections as a pro-inflammatory response, and infection models have also been used to determine activity following viral and bacterial infections in the chicken [29-31].

As shown in Figure-4, there was upregulation of IL6 at the 15^th^ day but to lower extent compared to early stages, these finding and skewing of the immune response to specific humeral immune response are similar to the responses of mammals [32,33]. Similar findings of IL6 being highly upregulated in HPAI H5N1 virus-infected chicken cells were reported by Kaiser *et al*. [34]. Elevation of IL6 has been observed in influenza-infected humans, primates, and ferrets, which appear to correlate with symptom severity [35,36]. IL-6 is a Th2 cytokine and induces antibody production in B cells, and promotes T cell activation and differentiation [37].

Expression of IFNs and pro-inflammatory cytokines influences both viral clearance and clinical disease presentation. In this study, results showed marked upregulation for the IFN γ in all vaccinated groups compared to the non-vaccinated group as shown in Figure-5. While at the 21 days postvaccination, there was a marked decrease in IFN-γ level in all formulated vaccines. A study found strong upregulation of IFN-γ mRNA in the lung and bursa of ducks but not chicken following infection with a LPAI H7N1 virus [38]. It is possible that IFN-γ could be important in protection against virulent influenza infection in avian hosts which permits further studies.

Collectively, the results showed that values of cellular immune response at later stages came in agreement with others [39] who stated that, once the humoral immune response becomes established; there is a corresponding decrease in the cellular immune response.

The performance of adjuvant vaccines not be evaluated only by their early response but also the level and duration of humoral immune response. Those parameters were investigated for each of the prepared vaccines by monitoring antibodies in the sera collected from vaccinated groups up to 20 WPV.

Anti-H9 serological evidence of experimentally vaccinated chicks against LPAI H9N2 in both monovalent vaccine and trivalent vaccine as shown in Figure-3 increased mean log2 HI antibody titer from the 3^rd^ WPV, respectively, then reached the highest HI antibody titer at the 8^th^ WPV, and then declined gradually to reach the lowest HI antibody titer at the 22^th^ WPV [40]. Reports showed that all vaccinated chickens with AI and AI+ND vaccines demonstrated high titers when tested by HI using homologous H9N2 antigen.

**Figure-6:**
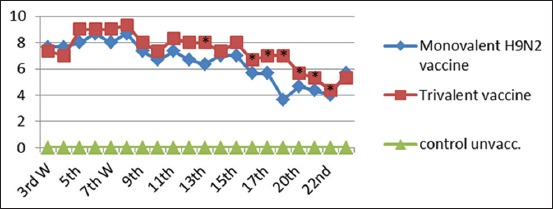
Mean anti-(H5N1) hemagglutination inhibition antibody titers in vaccinated chickens with monovalent avian influenza (AI) (H5N1) vaccine and trivalent AI (H9N2, H5N1)+Newcastle disease. *Significant difference at p<0.05 using Duncan’s multiple range tests.

HI serology of the prepared monovalent H5N1 and combined trivalent vaccine against H5N1 antigen as shown in Figure-6 revealed that Chickens in different vaccinated groups showed increased mean Log2 HI antibody from the 3^rd^ WPV, then reached the highest HI antibody titer (9 Log2) at the 8^th^ WPV for both vaccines, and then declined gradually to reach the lowest HI antibody titer at the 22^nd^ WPV. These findings suggested that the reassortant HPAI H5N1 viruses were avirulent and highly immunogenic [41]. It was noticed that the mean value of HI titer in the combined trivalent vaccine was significantly higher than monovalent vaccine in the 9^th^, 12^th^, 14^th^, 15^th^, 16^th^, 18^th^, and 22^nd^ WPV (p≤0.05), these results are in accordance with the study of El Sayed *et al.*, [42] who made a bivalent vaccine of NDV and H5N1 that give higher HI titers than the monovalent vaccines.

**Figure-7:**
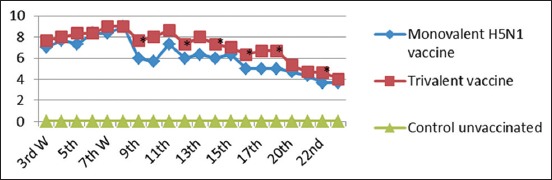
Mean Newcastle disease virus (NDV) - hemagglutination inhibition antibody titers in vaccinated chickens with monovalent NDV vaccine and trivalent avian influenza (H9N2, H5N1) + Newcastle disease. *Significant difference at p<0.05 using Duncan’s multiple range tests.

Detectable ND antibodies were detected in vaccinated chickens vaccinated with inactivated lasota NDV vaccine and combined trivalent AI (H9N2, H5N1) -ND vaccine with Montanide 71 adjuvant showed increased mean log2 HI antibody titer (6.33 log2 and 8.33 log2) from the 3^rd^ week post vaccination (WPV), then reached the highest HI antibody titer (9 log2 and 10.67 log2) at the 8^th^ WPV respectively then declined gradually to reach the lowest HI antibody titer (4.33 log2 and 5 log2) at the 22^th^ WPV as shown in Figure-7.

The fact that highest level of both cellular and humoral responses was conferred by the trivalent vaccine supports that there is an important factor contributes to the ability to confer immunity which is antigenic mass [5].

Challenge results showed severe clinical signs with 100% mortality in the control unvaccinated group post challenge. At the same time, 90% of the vaccinated chickens were protected from mortality and showed no clinical signs of HPAI infection for both monovalent H5N1 and trivalent vaccinated groups. Shedding test was carried out at the 3, 5, and 7 DPC from oropharyngeal swabs revealed that both monovalent H5N1 vaccine and trivalent vaccine were able to reduce the shedding of H5N1 virus. There was no viral shedding at 7 DPC. To examine the protective efficacy against H9N2, shedding level detection was carried out at 3, 5, and 7 DPC from oropharyngeal swabs revealed that both monovalent H9N2 vaccine and trivalent vaccine reduced H9N2 virus shedding. Sera from the control group were negative by HI (Table-2). This fulfills the OIE (2012) [43] requirements concerning the evaluation of vaccines by challenge test as any vaccine candidate must reduce the shed virus compared with group that receive challenge virus only (positive control group).

**Table-2:** Protective effectiveness of monovalent AI subtypes H9N2 and combined vaccine against H9N2.

Chicken group	Mean AB titter in HI test		Number of the shedding bird/total number of virus isolation from oropharyngeal (mean EID 50/ml)			Amount of dead birds/amount of birds in experiment	Protection %
	
	1 WPC	2 WPC	3^rd^ DPC	5^th^ DPC	7^th^ DPC		
Monovalent H9N2	7.5	8	(1/10)=1.0	(0/10)	0/10	1/10	90
Trivalent vaccine	7.7	9	0/10	0/10	0/10	1/10	90
Control unvaccinated	0	0	(10/10)=5.5	6	5	4/10	60

WPC=Week post challenge, DPC=Days post challenge, EID=Egg infective dose, HI=Hemagglutination inhibition, AI=Avian influenza

On the other hand, protection percent of the prepared vaccines against challenge with NDV was 100% in both monovalent NDV and trivalent vaccines confirming the potency and efficacy of the prepared vaccines (Table-3).

**Table-3:** Protective effectiveness of single NDV vaccine and combined vaccine against challenge with virulent NDV.

Birds	Mean Ab titer in HI test (Log2)		Amount of dead birds/amount of birds in experiment	Protection %

	1 WPC	2 WPC		
Monovalent NDV vaccine	8	9.5	0/10	100
Trivalent vaccine	8.5	10.5	0/10	100
Control	0	0	10/10	0

WPC=Weeks post challenge, NDV=Newcastle disease virus, HI=Hemagglutination inhibition

## Conclusion

The prepared trivalent vaccine candidate against NDV, H5N1, and H9N2 was as efficacious as monovalent counterparts, induced high titers of H5N1-, H9N2-, and NDV-specific antibodies and reduced H5N1 and H9N2 viral shedding.

Multivalent vaccines offer a number of practical advantages over monovalent vaccines; first advantage of multivalent vaccine is the fewer vaccinations required to mount an effective protection against several diseases, second is the reduced stress for the worker and the birds.

## Authors’ Contributions

This work is a part of ZMA’s PhD thesis supervised by AAEE, HAH, and MAEH. ZMA: Conducted the laboratory animal experimental work and drafted and revised the manuscript. MAMEH: Shared in design of the experimental work and followed up the practical part of the research. HAH: Set the design, supervised the work, drafted, and revised the manuscript. BMA: Analyzed the data and drafted and revised the manuscript. AAE: Conceived the study, set the design and supervised the work. All authors have revised and approved the final manuscript.
